# A serial mediating effect of perceived family support on psychological well-being

**DOI:** 10.1186/s12889-024-18476-z

**Published:** 2024-04-02

**Authors:** Jing An, Xuanyu Zhu, Zhan Shi, Jinlong An

**Affiliations:** 1 School of Management, Nanjing University of Posts and Telecommunications, 210003 Nanjing, Jiangsu Province China; 2 First People’s Hospital of Changshu City, Hospital Affiliated to Soochow University, Changshu, Jiangsu Province China

**Keywords:** Perceived family support, Psychological well-being, Serial mediating, Emotional well-being, Social well-being

## Abstract

Family has a significant impact on individual mental health. Based on social support theory, family system theory and the Mental Health Continuum Short Form (MHC-SF), this research constructed a model of the pathway of perceived family support on psychological well-being and the results empirically clarified that perceived family support has a significant positive relationship with emotional well-being, social well-being, and psychological well-being (*P* < 0.001). Emotional well-being positively influences social well-being and psychological well-being (*P* < 0.001). Social well-being positively affects psychological well-being (*P* < 0.001). There were direct mediating effects of emotional well-being (13.45%), direct mediating effects of social well-being (32.82%) and a serial mediating effect (28.07%) between perceived family support and psychological well-being (*P* < 0.001).

## Introduction

Perceived family support mainly refers to how an individual perceives the assistance received from other family members, such as parents. Numerous studies have demonstrated the significant impact of life stressors on mental health and overall well-being. Additionally, various social and personal resources, including social support, play a crucial role in influencing these outcomes [1]. There has been a pervasive consensus in the opinion that supportive interaction plays an indispensable role in shaping and maintaining an individual’s health and well-being. As a crucial element of perceived social support, perceived family support is a significant factor in promoting mental health. Prior research has provided evidence indicating that individuals who perceive a greater level of social support experience a range of positive outcomes which include increased positive emotions, improved physical and mental health, enhanced social relationships, a more optimistic perspective on life, and attainment of a higher level of subjective well-being [2]. Conversely, people who perceive low social support experience negative consequences like long-lasting advertisements [3], low life satisfaction, loneliness and depression [4–6], poor health [2], and drug abuse [7].

Subjective well-being is a major topic in the existing study on well-being. Ed Diener’s article in Psychological Bulletin [8] marked a significant contribution to the emerging field of subjective well-being (SWB), which focused on individuals’ subjective assessments of their own lives [9]. Additionally, Diener proposed that subjective well-being consists of two types of components, a cognitive judgment of one’s overall level of life satisfaction and affective experiences, reflecting people’s positive and negative emotional reactions to their lives [9]. Therefore, widespread studies have explained subjective well-being in terms of life satisfaction (LS), positive affect (PA) and negative affect (NA). This study also aims to investigate the underlying interaction mechanisms in well-being. As a result, Keyes’s linked research hypotheses on well-being were selected for this research. According to the related research of Keyes, well-being has different priorities under different streams, which are the hedonic stream and the eudaemonic stream. The hedonic stream defines mental health as “people’s attitude toward life, maintaining positive and healthy emotions in the face of life’s challenges, and the balance between negative and positive emotions” [10]. In contrast, the conceptualization of psychological and social well-being reflects the eudaemonic perspective, which focuses on how people view their functions in life and holds that mental health is the potential in human life [11] and people’s sense of social belonging or social function [12].

Previous studies showed that perceived family support could provide emotional support, information support and substantive support, and help people build self-confidence, self-esteem and self-regulation ability [2, 7]. However, there is still a lack of comprehensive understanding of how perceived family support affects mental health and well-being. Thus, the objective of this research is to investigate the pathway through which perceived family support influences psychological well-being and uncover the underlying mechanisms linking mental health and overall well-being. We aim to gain a deep understanding of the relationship among perceived family support, emotional well-being, social well-being, and psychological well-being, and provide an empirical basis for formulating psychological intervention measures and providing support strategies, to help people improve their psychological well-being.

## Theories and hypotheses

### Theoretical basis

Social Support Theory explains the influence of interpersonal relationships on individual well-being and adaptability [13]. It implies that social support is a combination of all types of actual or perceived help and resources obtained by people when under stress, dealing with issues, or adapting to environmental changes. The formulation of social support theory is grounded in the understanding of social support and its positive influence on human health and well-being. Research findings have indicated that social support can improve people’s mental health, reduce stress, promote physical health, improve individual adaptability and increase life satisfaction [14]. The development of social support theory also includes research on the sources, influencing factors and measurement methods of social support. Researchers found that social support can come from different interpersonal relationships and social networks, such as family, friends, colleagues, neighbors and community organizations [15]. In addition, the size, structure and quality of an individual’s social support network will also have an impact on their well-being and adaptability [16]. Intervention and research based on social support theory can help people better understand and promote the positive impact of social support on individual health and happiness, and then improve individual quality of life and well-being.

Family Systems Theory is a theoretical framework for family and individual development, which emphasizes the interdependence, mutual influence and interaction among family members. Based on this theory, family is conceptualized as an interconnected system, wherein the dynamics and interactions among family members exert a significant influence on individual behavior, emotion and development. The origin of family system theory can be traced back to the 1950s, which was put forward by psychologist Murray Bowen [17]. Bowen put forward the family system theory as a framework to explain mental illness and individual problems and combined it with the concept of *Differentiation of Self*. Subsequently, many researchers and clinical experts further developed and expanded the family system theory [18].

By analyzing the social support theory and family systems theory, we find that both theories emphasize the concepts of interaction, reciprocity and systematization [18, 19], the family is regarded as a system in society, in which family members are interrelated, influence each other and form a dynamic balance. Family members’ conduct and emotions form a cyclical pattern in the family system, which means that one person’s behavior impacts other people’s behavior, which in turn affects one’s behavior.

### Perceived family support and psychological well-being

According to the family stress model, in the family environment, people often encounter stressful events [20], which need to consume individual self-control resources to complete tasks. However, self-control resources are limited, and when resources are exhausted, the performance of subsequent tasks will decline. As a social resource, family support can be used as a resource supplement for self-control, helping us to adjust our state and face life positively. Therefore, perceived family support plays a vital role in improving emotional well-being. Based on the theory of social support, a supportive and nurturing family environment can provide security, love and acceptance [21], which are very important for emotional development, emotional stability and overall happiness. Family members can provide emotional support, understanding and encouragement to each other to help people cope with stress, anxiety and other emotional difficulties [7, 22]. Through fostering open communication, practicing active listening, and displaying empathy, families can establish a nurturing environment where individuals feel safe to express their emotions and seek solace [23], which also confirms the specific content of family system theory. Perceived family support helps to strengthen social relations by promoting positive interactions and relationships within families. These connections can extend beyond immediate families and have a positive impact on wider social networks and communities. Overall, perceived family support contributes significantly to social well-being by providing emotional, financial and practical help [24].

Perceived family support (PFS) provides a sense of belonging, love and acceptance, resulting in enhanced mental health and a stronger social support network. Active participation of family members in family interaction helps to improve their well-being [25]. When people get support from family members, it will exert a beneficial influence on their mental health and overall psychological well-being, which helps people feel more capable and potential, and reduces depression, stress and anxiety [7] also promotes security and confidence in facing life challenges and stimulates potential in life.

Therefore, the following assumptions are put forward:

#### H1

*Perceived family support has a significant positive relationship with emotional well-being.*

#### H2

*Perceived family support has a significant positive relationship with social well-being.*

#### H3

*Perceived family support has a significant positive relationship with psychological well-being.*

### Mental health and well-being

In Keyes’s research, emotional well-being (EWB), social well-being (SWB) and psychological well-being (PWB) jointly explain mental health and well-being [10]. When people experience positive emotions such as happiness, joy and satisfaction, it improves their overall psychological well-being and helps to gain a sense of accomplishment, resilience and a positive outlook on life. Besides, emotional well-being also plays a vital role in managing stress, anxiety and negative emotions. People with high emotional well-being are more able to cope with challenges and adversity, show stronger emotional resilience [26], and are more effective problem-solving abilities, have healthier interpersonal relationships and higher self-esteem levels [22]. And emotional well-being promotes self-awareness and self-regulation so that people can effectively understand and manage their emotions [27]. This ability to regulate emotions contributes to better mental health outcomes, including reduced symptoms of depression and anxiety.

In addition, emotional well-being contributes to the formation and maintenance of healthy and meaningful relationships. Emotion Regulation Theory [28] points out that individuals’ ability to recognize and regulate their own emotions will affect their emotional communication and relationship with others. Concurrently, the theory of Emotional Intelligence suggests that possessing the capacity to perceive, comprehend, and regulate one’s own and others’ emotions is crucial for facilitating effective interpersonal interactions and managing emotions adeptly [29]. Therefore, when people have positive emotions, they are more likely to have warm, supportive and empathetic interactions with others. When people maintain emotional balance and coordinate with their own emotions, they can better understand and deal with other people’s emotions, and can better express themselves clearly, actively listen to others and constructively manage conflicts. It facilitates effective communication, promotes understanding, makes them approachable and reduces misunderstandings or conflicts in social interactions and promotes deeper ties, empathy and social support, thus strengthening social ties. And it is more likely to arouse positive reactions from others, which leads to more pleasant and harmonious social interaction. It, in turn, helps to create a positive social atmosphere and promote social well-being.

Because of the innate demand of human beings for social relations and the profound influence of social interaction on mental health, there is also a strong connection between social well-being and psychological well-being. Social well-being provides people with a sense of belonging and connection to others. Empirical investigations demonstrated that self-perceived social integration acted as a protective factor for the subjective well-being of immigrants, contributing to higher levels of life satisfaction and lower levels of loneliness [30]. When people feel a social connection, support and acceptance through social networks, they will enhance their self-esteem, self-worth and overall psychological well-being. Perceived belonging to a supportive social group (such as family) can buffer loneliness, isolation and depression [7]. As mentioned in the social support theory, positive social interaction is very important for individual psychological well-being [13]. Social well-being involves positive and satisfying social interactions, such as spending time with relatives, participating in conversations, attending social gatherings, etc., which can arouse positive emotions and increase well-being. Generally speaking, social well-being provides a supportive environment for cultivating psychological well-being, which meets the demands of human beings for connection, belonging and support, provides opportunities for personal growth and realization, promotes positive social interaction and stimulates cognitive function. These factors together promote the enhancement of psychological well-being and the improvement of quality of life.

Therefore, the following assumptions are put forward:

#### H4

*Emotional well-being has a significant positive impact on psychological well-being.*

#### H5

*Social well-being has a significant positive impact on psychological well-being.*

#### H6

*Emotional well-being has a significant positive impact on social well-being.*

### Emotional well-being and social well-being as mediators

Perceived family support may have indirect relationship with psychological well-being by affecting emotional and social interactions. It suggests that when people perceive family support, they may be more likely to experience positive emotions, such as happiness and satisfaction, which are beneficial for psychological well-being [22]. Additionally, perceived family support may also have indirect relationship with psychological well-being by facilitating the development of social interactions and relationships [25]. People perceive more social support and a sense of belonging, which may also have a positive relationship with psychological well-being. Meanwhile, emotional well-being may have a significant positive influence on social well-being [29].

Therefore, the following assumptions are put forward:

#### H7

*There were direct mediating effects of emotional well-being, direct mediating effects of social well-being and a serial mediating effect between perceived family support and psychological well-being.*

**Fig. 1:**
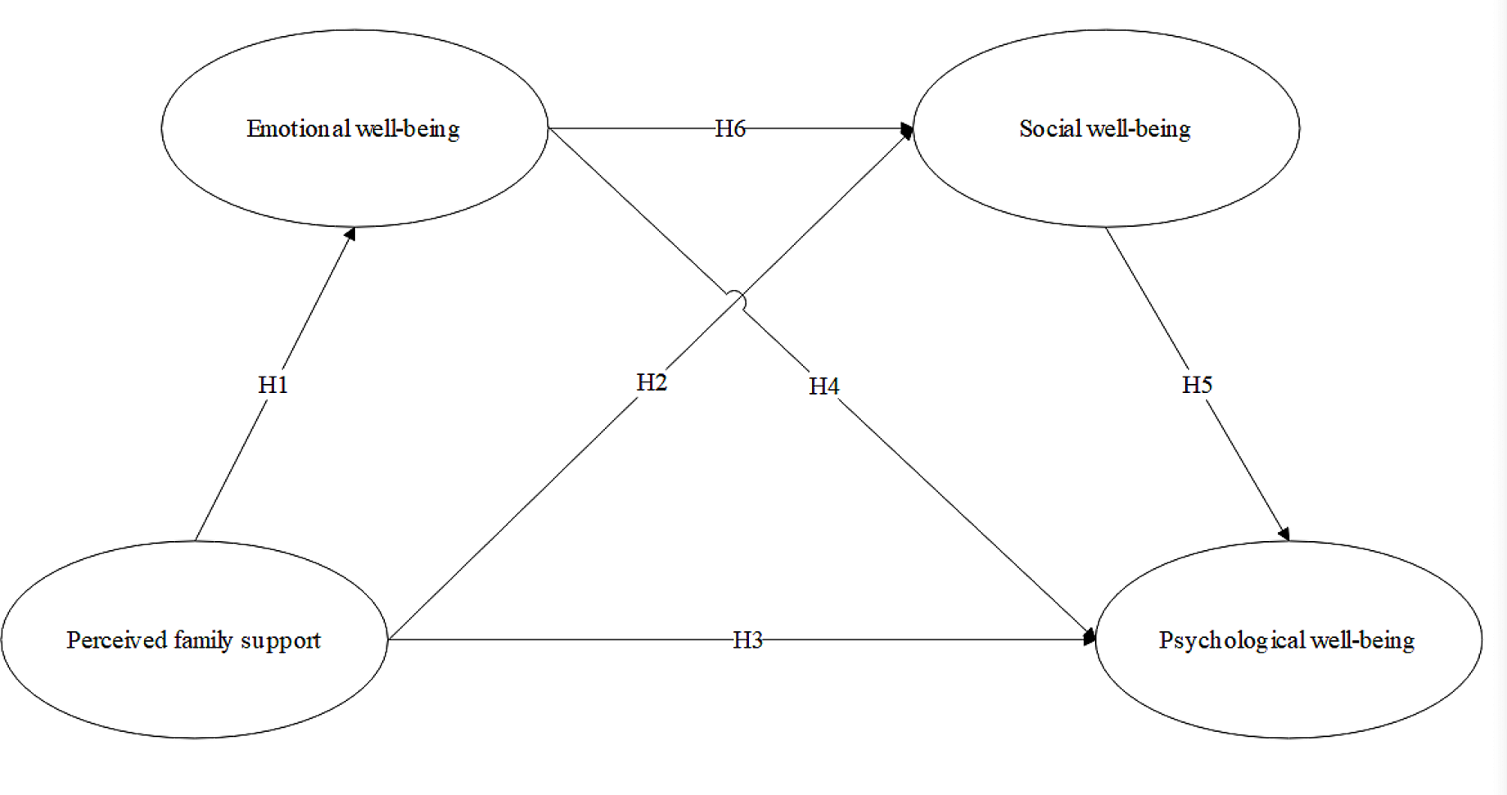
Research model

The research model is shown in Fig. 1.

## Method

### Data collection

The questionnaire survey was distributed through the online socializing platform including WeChat and Tencent QQ from October 2020 to June 2021. A total of 894 questionnaires were collected in this survey. The respondents were mainly undergraduates and postgraduates from several universities in China. To increase the diversity of participants, we also collected responses from overseas Chinese college students living abroad. Ethical approval for this research has been obtained from the Ethics Committee of University. Each participant was informed of the survey and filled out the informed consent.

### Measure

The questions for measuring perceived family support come from the multidimensional scale of the perceived social support project [31], “My family really tries to help me.“, “I can talk about my problems with my family.” And “My family is willing to help me make decisions”. The 7-point Likert scale was used (1 = very disagreeable to 7 = very agreeable). Higher scores indicated better-perceived family support. In this study, the Cronbach’s α coefficient of this scale was 0.83.

This study used the Mental Health Continuum Short Form (MHC-SF) developed by Keyes, a self-reported questionnaire with 14 items. The MHC-SF evaluates mental health in emotional well-being (e.g. Self-perceived happiness), social well-being (e.g. Social identity) and psychological well-being (e.g. Personal preferences) and adopts a 6-point Likert scale (from 0 = ‘never ‘to 5 = ‘always’). In this study, emotional well-being, social well-being and psychological well-being were measured by their corresponding subscales. Higher scores indicate better mental health. Many studies had measured and evaluated the structure, reliability, convergence validity [32] and gender invariance [33] of MHC-SF in different cultures and countries, including Italy [34], Netherlands [32, 35], South Africa [10], Poland [36], Spain [37] and China [38]. Some scholars used MHC-SF to predict individual mental health and well-being and explored possible influencing factors and found ways to improve mental health [39]. Cavioni and other scholars used structural equation modeling to assess whether relationships at school with teachers and peers, and life satisfaction predicted mental health in a large sample of adolescents [33]. Giannopoulos and Vella-Brodrick found that positive intervention and happiness orientation have an impact on well-being [40]. Karaś et al. examined the relationships between identity processes and well-being across various life domains [41]. In this study, the Cronbach’s α coefficient of emotional well-being, social well-being and psychological well-being subscales was 0.911, 0.873, and 0.933.

### Procedure

The data analysis included model fitting, hypothesis testing, and mediating effect testing. The mediating effect tests involved two direct mediating effect analyses and a serial mediating effect analysis to examine whether there was a mediating effect between perceived family support and psychological well-being.

### Results and data analysis

#### Reliability analysis

Cronbach’s α coefficient is used as the reliability analysis index in this study, α greater than 0.8 indicates that the scale has good reliability. Since the lowest α for a variable was 0.830, the reliability of the scale met the requirements and the α after deletion was less than the standardized α in all variables, so the reliability of the scale had good reliability.

### Confirmatory factor analysis and common method bias test

The structural validity results of confirmatory factor analysis (CFA) model A, including all items are shown in the Table 1. The χ2/df of model A was 4.057 > 3, which did not meet the requirement. The validity of the current instruments arrangement scale was not valid, so it was necessary to adjust. First of all, we chose to remove the items SO1 and SO2 with an estimate less than or equal to 0.7 and then carried out a CFA of model B(removing the items SO1 and SO2). The results were as shown in the table, and the χ2/df of model B was 3.559 > 3, and the validity was still not up to standard. We observed the covariance between the residuals of each item and found that the covariance between the residuals of PSY3 and the residuals of latent variables social, psychological and PSY6 was greater than 20, which had a strong positive correlation. Therefore, the PSY3 item was removed and analyzed again, and the results of CFA model C (removing the items SO1, SO2 and PSY3) were obtained. The χ2/df of model C was 2.973 < 3, and RMSA was 0.047 < 0.05. Therefore, the scale had good structural validity (see Table 1).

**Table 1:** CFA results

model	χ2/df	RMSEA	SRMR	TLI	CFI	RMSEA
Model A	4.057	0.059	0.030	0.964	0.970	0.059
Model B	3.559	0.056	0.024	0.974	0.980	0.056
Model C	2.973	0.047	0.022	0.981	0.985	0.047

In this study, confirmatory factor analysis (CFA) was used to test the validity of the questionnaire. The convergence validity of each variable in the model was evaluated by Estimate, Composite Reliability (CR) and Average Variance Extracted (AVE). As shown in Table 2, the standardized estimates of the observed variables associated with the four latent variables in the research model exceeded the recommended threshold of 0.5, demonstrating statistical significance and indicating a strong convergence of the questionnaire used in this study. The CR of the four latent variables was between 0.834 and 0.924, which was greater than 0.7, indicating that the measurement model of this study had good internal consistency; The AVE of the latent variables were between 0.627 and 0.775, which were all greater than the recommended value of 0.5, indicating that the measurement model had good convergence. The square root of the Average Variance Extracted (AVE) for each latent variable exceeded the correlation coefficient between that particular latent variable and other latent variables (see Table 3). This observation suggests that the measurement model demonstrated favorable levels of differential validity.

**Table 2:** Estimates, combined reliability and average variance extraction

path		Estimate	S.E.	CR	P	AVE	CR
FAM2 <--	PFS	0.776				0.627	0.834
FAM3 <--	PFS	0.8761	0.0474	24.1123	***		
FAM1 <--	PFS	0.7159	0.0445	21.0017	***		
EM1 <--	EWB	0.8621				0.775	0.912
EM2 <--	EWB	0.9048	0.0304	36.321	***		
EM3 <--	EWB	0.8729	0.0318	34.2667	***		
PSY1 <--	PWB	0.8167				0.710	0.924
PSY2 <--	PWB	0.8502	0.033	30.3374	***		
PSY6 <--	PWB	0.8604	0.0352	30.8894	***		
PSY4 <--	PWB	0.8273	0.0333	29.1375	***		
PSY5 <--	PWB	0.8574	0.0341	30.7279	***		
SO3 <--	SWB	0.8204	0.0379	28.0973	***	0.707	0.879
SO5 <--	SWB	0.8771	0.0356	30.648	***		
SO4 <--	SWB	0.8244					

**Table 3:** Differential validity results

	PFS	EWB	PWB	SWB
PFS	0.792			
EWB	0.5508	0.880		
PWB	0.5974	0.7891	0.843	
SWB	0.5576	0.7839	0.8093	0.841

*Note* The italics on the diagonal represent the square root of AVE

Based on Podsakoff’s suggestion, the model C with homologous bias fit well (χ2/df = 2. 417, RESEA = 0. 039, SRMR = 0. 016, TCL = 0. 986, CFI = 0. 992). Compared with the original model C (χ2/df = 2. 973, RESEA = 0. 047, SRMR = 0. 022, TCL = 0. 981, CFI = 0. 985) (see Table 4), the variation of RMSEA and SRMR were less than 0.01 (the variation value is less than 0.05), and the variation of CFI and TLI were less than 0.01 (the difference is less than 0.1), which indicated that the model C with common method factor control have not been improved obviously, showing there was no serious homologous deviation of variables.

**Table 4:** Common method deviation test

model	χ2/df	RMSEA	SRMR	TLI	CFI
Model C	2.973	0.047	0.022	0.981	0.985
Model C with homology bias	2.417	0.039	0.016	0.986	0.992

### Characteristics of participants

The percentage of male and female respondents is 50.4% and 49.6% respectively. Their ages are mainly between 18 and 22 (62.9%) and 22–30 (25.5%) respectively. The majority of respondents are college students (71.7%). Almost 90% of them are online for more than two hours daily (see Table 5).

**Table 5:** Frequency analysis of demographic variables

Variable	Classification	Frequency	Percentage (%)	SD
Gender	Female	443	49.6	0.5
	Male	451	50.4	
Age	18 ~ 22 years old	562	62.9	0.748
	23 ~ 30 years old	228	25.5	
	31 ~ 45 years old	87	9.7	
	Under 45 years old	17	1.9	
Education level	Junior high school	6	0.7	0.651
	High school	55	6.2	
	Undergraduate	641	71.7	
	Master	145	16.2	
	PhD	47	5.3	
Education level of parents	Junior high school	371	41.5	0.919
	High school	305	34.1	
	Undergraduate	184	20.6	
	Master	19	2.1	
	PhD	15	1.7	
Annual family income	Less than 50,000 RMB	183	20.5	1.023
	50,000-100,000 RMB	287	32.1	
	100,000-200,000 RMB	251	28.1	
	More than 200,000 RMB	173	19.3	
Daily online time	Less than 2 h	91	10.2	0.978
	2–4 h	282	31.5	
	4–6 h	265	29.6	
	More than 6 h	256	28.6	
Years of surfing	Less than 3 years	49	5.5	0.922
	3–6 years	236	26.4	
	6–10 years	282	31.5	
	More than 10 years	327	36.6	

### Model fitting

AMOS was used to test the model fitting degree. It is evident that all the fitting coefficient of the model (χ2/ df = 2.973 < 3, RMSEA = 0.047 < 0.05, CFI = 0.985 > 0.9, TLI = 0.981 > 0.9, IFI = 0.985 > 0.9) reach the required standard, indicating that the model possesses the ability to adapt effectively.

The absolute values of critical ratios C.R. were all greater than 1.96 (see Table 6). The probability values of significance of all pathways were significant (*P* < 0.001), and the normalized coefficient was greater than 0, indicating that H1-H6 were supported.

**Table 6:** Hypothesis test results

Hypothesis	Path	Standardization coefficient	S.E.	C.R.	P
H1	EWB <---PFS	0.5508	0.0334	14.527	***
H2	SWB <----PFS	0.1807	0.0314	5.2811	***
H3	PWB <----PFS	0.1532	0.0273	5.123	***
H4	PWB <----EWB	0.356	0.0446	8.2685	***
H5	PWB <----SWB	0.4448	0.045	9.8349	***
H6	SWB <----EWB	0.6844	0.0395	18.0341	***

### Mediating effect test

Preacher and Hayes’ Bootstrapping mediating effect [42] test method is adopted, which provides a 95% confidence interval estimation of a mediating effect. If the upper and lower bound estimation of the interval contains 0, the mediating effect is not significant, and if the interval estimation does not contain 0, the mediating effect is significant. Three indirect effects and one direct effect between perceived family support and psychological well-being existed significantly (*P* < 0.01), indicating H7 was supported. As the social well-being was a mediating variable, the influence of “perceived family support” on “psychological well-being” accounted for 13.45% of the total effect. When emotional well-being was a mediating variable, the influence effect of “perceived family support” on “psychological well-being” accounted for 32.82% of the total effect. When emotional well-being and social well-being were used as mediating variables, the influence effect of “perceived family support” on “psychological well-being” accounted for 28.07% of the total effect. The direct effect of “perceived family support” on “psychological well-being” accounted for 25.65% of the total effect (see Table 7).

**Table 7:** Mediating test results

Parameter	Estimate	Lower	Upper	P	Percentage
Perceived Family Support-> Social Well-being-> Psychological Well-being	0.0734	0.0407	0.1195	0.0006	13.45%
Perceived family support-> emotional well-being-> psychological well-being	0.179	0.1211	0.253	0.0006	32.82%
Perceived family support-> emotional well-being-> social well-being-> psychological well-being	0.153	0.1076	0.2094	0.0007	28.07%
Perceived family support-> psychological well-being	0.1398	0.0751	0.2154	0.0006	25.65%
Total	0.5452	0.4716	0.6269	0.0006	

## Discussion

This study aims to examine the predictive role of perceived family support on psychological well-being. The results showed that perceived family support could well predict the well-being of mental health. The presence of some mediating factors between perceived family support and psychological well-being, including the direct mediating between emotional well-being and social well-being, and the serial mediating between emotional well-being and social well-being. It indicated a mediating moderation effect between perceived family support and psychological well-being.

### Associations between perceived family support and psychological well-being

As the results showed, the perceived family support, the selfless help of the family, the decision support of the family and whether the family is the object of talking, were positively correlated with emotional well-being, social well-being and psychological well-being. Hypotheses 1, 2 and 3 were supported, which was the same as the previous research results [43–45]. Chinese researchers found that maternal support had a positive effect on adolescents’ subjective well-being [43]. Children’s mental framework was affected by maternal and paternal warmth and hostility, thus affecting children’s executive ability [44]. Moreover, some results showed that among parents who have children with chronic kidney disease and patients who have multiple sclerosis, family social support help them to adaptation for a better quality of life [46–47]. The perception of selfless help and decision support from families increased the happiness of people, which also reflected the specific content of social support theory [13]. Social support theory held the view that when people faced pressure and challenges, getting emotion, information and practical support from others could have a positive impact on their emotional health. As a major social support network, the family provided emotional support, understanding and assistance among family members, which could enhance an individual’s emotional health. Considering that family members could be the object of talking about problems would also increase their psychological well-being, which showed the interaction and interdependence among family members emphasized by the family system theory [17]. Support and cooperation among family members could promote family harmony and stability, and help people adjust their emotions and feel healthy. When the family tried their best to help family members and was willing to participate in the members’ decision-making process, the members would feel valued and supported, thus improving their mental health.

### The mediation effect between perceived family support and psychological well-being

The study discovered that emotional well-being acted as a mediating factor between perceived family support and psychological well-being, indicating that perceived family support had the potential to enhance psychological well-being by fostering individual emotional well-being. This was consistent with the results of previous study findings that perceived family support positively predicted emotional health and emotional health positively predicted mental health [23]. LaMontagne and other scholars proposed that adolescent emotion regulation mediated the relationship between family conflict and adolescent depression [7]. Perceived family support could promote individuals’ psychological well-being by positively affecting their emotional well-being. When people perceived family support, they might feel more emotional satisfaction and happiness, which in turn had a positive impact on their psychological state. This mediating process illustrated the bridge between emotional well-being and perceived family support and psychological well-being.

As shown in this study, social well-being played a mediating role in the relationship between perceived family support and psychological well-being, and perceived family support facilitated psychological well-being by enhancing an individual’s social well-being. This was consistent with previous findings that perceived family support positively predicted social health and social health positively predicted mental health. The study focusing on adolescents aged 13–18 years in Ghana’s Upper West Region observed that even when considering personal characteristics, there were variations in the relationship between family sense of belonging, autonomy support, control, and social support, and self-reported life satisfaction and happiness among adolescents [24]. Social well-being involved subjective feelings and satisfaction of people in social interaction. When people perceived support from their families, they might be more likely to establish and maintain good social relations, obtain social support and feel the satisfaction of social connections. Therefore, perceived family support could improve the psychological well-being of people by increasing their social well-being.

We found that emotional well-being and social well-being played a serial mediating role in the relationship between perceived family support and psychological well-being. Perceived family support would increase social well-being by increasing emotional well-being, and finally lead to an increase in psychological well-being. Perceived family support affected emotional well-being and social well-being, while emotional well-being and social well-being had an impact on psychological well-being. The increase in emotional well-being led to an increase in social well-being, which was also proved by the study of Emotional Intelligence [29]. Perceived family support had a positive impact on emotional well-being. When people perceived a higher level of support from their families, they were more likely to experience positive emotions, lower levels of distress, and achieved a greater degree of overall happiness. Emotional well-being covered happiness, satisfaction and positive emotional experience in daily life. Emotional well-being played a mediating role between perceived family support and social well-being. In addition, when people had a higher level of emotional well-being, they were more likely to participate in positive social interactions, establish and maintain supportive interpersonal relationships, and experience a sense of belonging and social connections. Moreover, social well-being played a mediating role between emotional well-being and psychological well-being. When people had a higher level of social well-being, they were more likely to have better psychological well-being, which included higher levels of self-esteem and lower levels of psychological distress. Social well-being provided people with a sense of support, recognition and social integration, thus positively affecting their overall psychological well-being. Perceived family support enhanced emotional well-being, which in turn enhanced social well-being and ultimately led to an increase in psychological well-being. The serial mediation process emphasized the importance of emotional well-being and social well-being, as the mediating factors of perceived family support affecting individual psychological well-being.

### Limitations and future studies

This study evaluated the predictive effect of perceived family support on psychological well-being in China, but there were some limitations. First of all, this study focused on emotional well-being and social well-being as mediating variables, but there might be other mediating variables in the actual situation, such as personal accomplishment and satisfaction. Therefore, attributing the mediating effect only to emotional well-being and social well-being might not fully explain the whole mediating process. Secondly, this kind of research was often based on cross-sectional data or longitudinal survey design, which made it difficult to determine the causal relationship. We couldn’t determine whether perceived family support directly led to changes in emotional well-being and social well-being, or whether other factors affected these variables at the same time. Therefore, more long-term follow-up studies or experimental designs were necessary. Thirdly, the study relied on the self-reported data of the subjects, and there was the possibility of self-reported bias. People might be affected by recall bias, social expectation and self-presentation, which led to subjectivity and bias in reporting perceived family support, emotional well-being and social well-being. Finally, in the study, there might be unconsidered variables, which might have an impact on the relationship between perceived family support, self-control of online time and happiness, such as individual personality traits and social support networks.

Future research should determine the causal relationship among perceived family support, self-control and happiness, and explore other possible mediating variables to more comprehensively understand the relationship among perceived family support, self-control and happiness, such as self-efficacy or friend support. Cross-cultural comparative studies can also be conducted to explore the relationship among perceived family support, self-control and happiness in different cultural backgrounds. Different cultural backgrounds may influence the relationship between these variables, so comparing the results under different cultures can increase the understanding and popularization of this field.

## Conclusion

This study provides a unique exploration of the impact of perceived family support on psychological well-being. The Mental Health Continuous-Short Form (MHC-SF) was utilized to measure and predict social, emotional, and psychological well-being, while the multidimensional scale of perceived social support was employed to assess perceived family support. The study investigates the relationship between perceived family support and psychological well-being, as well as the mediating effects of emotional well-being and social well-being. These findings may contribute valuable insights for future research in this field.

## Data Availability

The data sets used and analyzed in this study are available from the corresponding author on reasonable request.
